# α2δ-1 Signaling Drives Cell Death, Synaptogenesis, Circuit Reorganization, and Gabapentin-Mediated Neuroprotection in a Model of Insult-Induced Cortical Malformation

**DOI:** 10.1523/ENEURO.0316-17.2017

**Published:** 2017-11-06

**Authors:** Lauren A. Lau, Farzad Noubary, Dongqing Wang, Chris G. Dulla

**Affiliations:** 1 Department of Neuroscience, Tufts University School of Medicine, Boston, MA 02111; 2 Neuroscience Program, Tufts Sackler School of Biomedical Sciences, Boston, MA 02111; 3 The Institute for Clinical Research and Health Policy Studies, Tufts Medical Center, Boston, MA 02111; 4 Tufts Clinical and Translational Science Institute, Tufts University, Boston, MA 02111; 5 Department of Brain and Cognitive Sciences, Massachusetts Institute of Technology, Cambridge, MA 02139

**Keywords:** a2d-1, cell death, cortex, gabapentin, malformation, polymicrogyriax

## Abstract

Developmental cortical malformations (DCMs) result from pre- and perinatal insults, as well as genetic mutations. Hypoxia, viral infection, and traumatic injury are the most common environmental causes of DCMs, and are associated with the subsyndromes focal polymicrogyria and focal cortical dysplasia (FCD) Type IIId, both of which have a high incidence of epilepsy. Understanding the molecular signals that lead to the formation of a hyperexcitable network in DCMs is critical to devising novel treatment strategies. In a previous study using the freeze-lesion (FL) murine model of DCM, we found that levels of thrombospondin (TSP) and the calcium channel auxiliary subunit α2δ‐1 were elevated. TSP binds to α2δ‐1 to drive the formation of excitatory synapses during development, suggesting that overactivation of this pathway may lead to exuberant excitatory synaptogenesis and network hyperexcitability seen in DCMs. In that study, antagonizing TSP/α2δ‐1 signaling using the drug gabapentin (GBP) reduced many FL-induced pathologies. Here, we used mice with a genetic deletion of α2δ‐1 to determine how α2δ‐1 contributes to cell death, elevated excitatory synapse number, and in vitro network function after FL and to examine the molecular specificity of GBP’s effects. We identified a critical role for α2δ-1 in FL-induced pathologies and in mediating the neuroprotective effects of GBP. Interestingly, genetic deletion of α2δ-1 did not eliminate GBP’s effects on synaptogenesis, suggesting that GBP can have α2δ-1-independent effects. Taken together these studies suggests that inhibiting α2δ-1 signaling may have therapeutic promise to reduce cell death and network reorganization associated with insult-induced DCMs.

## Visual Abstract

**Figure:**
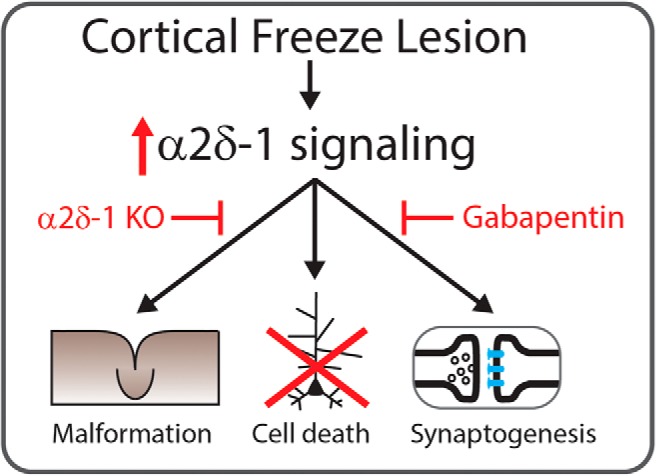

## Significance Statement

α2δ-1 signaling drives apoptotic cell death, anatomic reorganization, excitatory synaptogenesis, astrocytosis, and network hyperexcitability in a model of insult-induced cortical malformation known as freeze lesion (FL). This is the first direct demonstration of α2δ-1’s role in injury-induced cell death in the CNS, and suggests that α2δ-1 may be broadly involved in pathologic changes after neonatal brain insult. We also investigated the effects of gabapentin (GBP) in the absence of α2δ-1, a known site of drug action. Our studies show that genetic deletion of α2δ-1 eliminates GBP’s neuroprotective effects, confirming a specific site of action. We also find that GBP can reduce FL-induced increases in excitatory synapse number in α2δ-1 knock-out (KO) mice, indicating GBP has α2δ-1-independent anti-synaptogenic effects.

## Introduction

Developmental cortical malformations (DCMs) are characterized by an anatomically disorganized cortex and are associated with difficult to treat epilepsy (Crino, 2005; Takano et al., 2006). There are multiple DCM subsyndromes, including tuberous sclerosis, focal cortical dysplasia (FCD), and polymicrogyria. Both genetic and environmental causes of DCMs exist, with individual subsyndromes having their own etiology. These include mutations in genes responsible for neuronal proliferation, migration, and apoptosis (Barkovich et al., 2012), as well as neonatal insults including pre- and perinatal hypoxia, viral infection, and traumatic injury (Leventer et al., 2008; Blümcke et al., 2011). DCMs associated with neonatal insult (polymicrogyria and FCD Type IIId) may be more amenable to therapeutic intervention, as their precipitating insult is often rapidly clinically documented.

To better understand the molecular and cellular changes associated with early life insult-induced cortical network reorganization, we used the neonatal freeze-lesion (FL) model. FL recapitulates key features of polymicrogyria and FCD Type IIId (Luhmann, 2016) including insult-induced etiology, cell death, astrocytosis, anatomic reorganization, and spontaneous seizures (Sun et al., 2016; Williams et al., 2016). Additionally, the FL model allows study of the changes that occur between insult and network dysfunction in a stereotyped model. Briefly, on the day of birth (postnatal day 0, P0), a freezing probe is applied to the exposed skull, generating a hypoxic insult. This results in cell death and the formation of a microgyrus and a surrounding paramicrogyral zone (PMZ). The PMZ is hyperexcitable and generates epileptiform network activity beginning approximately two weeks after injury (Jacobs et al., 1996; Jacobs et al., 1999; Andresen et al., 2014). Identifying the molecular, cellular, and network level changes that lead to brain dysfunction after neonatal cortical insult are paramount to developing novel therapeutic approaches.

Recently, we reported that thrombospondin (TSP)/α2δ-1 signaling is increased in the cortex following FL (Andresen et al., 2014). TSP promotes excitatory synaptogenesis during development (Christopherson et al., 2005) by binding to α2δ-1 (Eroglu et al., 2009). α2δ-1 acts as an auxiliary subunit of voltage gated calcium channels, but its synaptogenic function appears to be independent of calcium channel association (Eroglu et al., 2009). Recent genetic studies have implicated α2δ-1 mutations in human polymicrogyria (Vergult et al., 2015). Of note, gabapentin (GBP) blocks the interaction of TSP and α2δ-1 and reduces α2δ-1-mediated excitatory synaptogenesis (Eroglu et al., 2009). There is reason to suspect that brain injury reactivates TSP/α2δ-1, leading to hyperexcitation. *In vivo* GBP treatment attenuates injury-induced increases in synaptogenesis (Li et al., 2012; Li et al., 2014; Takahashi et al., 2017) following adult brain injury, and reduces excitatory synaptic transmission and cortical hyperexcitability following FL (Andresen et al., 2014). Interestingly, GBP is also neuroprotective in models of spinal cord injury (Emmez et al., 2010; Kale et al., 2011), cortical trauma (Li et al., 2012) and stroke (Traa et al., 2008; Kim et al., 2010). It is unknown, however, whether α2δ-1 activity contributes to cell death or if GBP’s neuroprotective effects are mediated by its actions on α2δ-1.

In this study, we examined the role of α2δ-1 in FL-associated pathologies (cell death, cortical reorganization, astrocytosis, excessive synaptic excitation, and network hyperexcitability) and GBP-mediated neuroprotection. We used both pharmacological blockade and genetic deletion of α2δ-1 and found that both reduced apoptotic cell death following FL. Attenuating α2δ-1 signaling pharmacologically or genetically helped restore normal cortical structure following FL, reduced astrocyte reactivity, and attenuated FL-induced increases in excitatory synapse number and glutamatergic synaptic transmission. The vast majority of GBP’s effects were absent in mice lacking α2δ-1, confirming that GBP acts largely through α2δ-1. Our studies of synapse number, however, suggest GBP may also have α2δ-1-independent effects. Additionally, cortical hyperexcitability is only partially rescued in FL mice deficient in α2δ-1, consistent with other pathways contributing to dysfunction following neonatal cortical insult. To enhance the rigor of this study we also employed a linear mixed model statistical approach and found that GBP and α2δ-1 have robust effects on FL pathology. Overall, our study suggests that α2δ-1 may be involved in driving multiple pathologies associated with insult-induced DCMs, as well as in mediating the neuroprotective effects of GBP.

## Materials and Methods

### Animals

All guidelines of Tufts University’s Institutional Animal Care and Use Committee were followed. α2δ-1 knock-out (KO; α2δ-1^−/−^) animals were generated by crossing heterozygous (α2δ-1±) pairs. Wild-type (WT; α2δ-1^+/+^) littermates were used as controls. α2δ-1^−/−^ mice were a generous gift of Guoping Feng (Massachusetts Institute of Technology). Mice were maintained on a C57BL6/J background and back crossed for >20 generations. All experiments use mice from multiple litters to reduce litter to litter variability.

### FL

Experimental microgyri in primary somatosensory cortex (right hemisphere) were induced in P0 WT or α2δ-1 KO mouse pups by freeze lesioning as described previously (Andresen et al., 2014). Briefly, animals were anesthetized by hypothermia, an incision into the scalp was made, and a copper probe cooled to −50°C to −60°C was placed onto the exposed skull for 5 secs. Sham operated littermates were generated by leaving the probe at room temperature. After freeze-lesioning, the incision was closed using surgical glue, and pups were warmed and returned to the dam. All FL surgeries were performed by the same experimenter to reduce variability in the procedure, who was blinded to the genotype/treatment group.

### Drug treatments

For drug treatment experiments, mice were treated with once daily intraperitoneal injections of either GBP (200 mg/kg) or vehicle (sterile injection saline) from P1 to P7.

### Western blotting

Expression of α2δ-1 was analyzed via western blot. Cortex from adult α2δ-1 KO and WT littermates was dissected and homogenized using lysis buffer (0.2% SDS, 50 mM NaF, and 1 mM EDTA). Buffer (10 mM Tris, pH 8, 150 mM NaCl, 5 mM EDTA, 1% Triton X-100, 10 mM NaF, 2 mM Na_3_VO_4_, and 10 mM Na_4_P_2_O_7_) with 100× Halt protease inhibitor cocktail was added and samples were spun down for 15 min at 4°C. Loading buffer (10 μl 2-mercaptoethanol and 190 μl Laemmli buffer) was added to samples 1:1 and samples were heated to 70°C for 10 min. A total of 25 μg of protein was loaded onto a 10% SDS-PAGE gel and analyzed via electrophoresis. Protein was detected using antibodies for α2δ-1 (1:500, Abcam RRID:AB_303365), and anti-β-actin (1:1000, Abcam) was used to confirm equal loading. Protein bands were visualized using enhanced chemiluminescence and imaged with a LAS-3000 imaging system.

### Behavioral tests

Ten-week-old male mice were used for behavioral testing. Behavioral assays were performed in the Tufts Center for Neuroscience Research Animal Behavior Facility. Animals were tested on a battery o*f* tests, starting with the least stressful test (open field) and moving to more stressful tests (spontaneous alteration and rotarod) on subsequent days.

#### Open field test

Mice were individually placed into the center of a 40 × 40 cm open field apparatus with 16 × 16 equally spaced photocells (Hamilton-Kinder). The total distance traveled, and the amount of time spent in the center of the open field, as well as the total number of beam breaks, were measured during the 20-min test using MotorMonitor software (Hamilton-Kinder).

#### Spontaneous alteration

Mice were placed in a three arm T-maze. After being placed in the starting arm, the barrier was removed to allow the rodent free choice to enter either the left or right arm. After entering an arm, a barrier was placed to keep the animals in that arm for 30 s. The mouse was returned to the home cage for 1 min between trails and a total of 10 trials were performed. Generally, rodents will engage in exploratory behavior in the maze and alternate between the arms. The percentage of trials in which the animal spontaneously alternated arms was reported.

#### Rotarod

Motor coordination and balance was measured in the mice using an acceleration paradigm on a five position rotarod (ENV 577M, Med Associates). Mice were trained at a constant, slow speed of 14 rpm until they could successfully stay on the beam for 5 min. Testing was conducted using a fast acceleration paradigm (4−40 rpm over 5 min). Mice were tested over the course of three trials (30 min apart) and latency to fall and the RPM at which mice fall was reported.

### Electroencephalogram (EEG) recording

Naïve, 10-week-old WT and α2δ-1 KO littermates were implanted for EEG recording based on a Tufts Institutional Animal Care and Use Committee approved protocol. Briefly, animals were anesthetized (100 mg/kg ketamine and 10 mg/kg xylazine). An incision was made on the scalp, the surface of the skull was dried, and four burr holes were drilled with care to avoid puncturing the dura. Four 0.1" stainless steel screws (Pinnacle Technologies) were gently screwed into the drilled holes. A common reference electrode was placed anterior to the left side of the intraparietal bone, and two EEG electrodes were placed in the left front bone (for front/motor cortex) and in the right parietal bone (for somatosensory cortex). A separate ground electrode was also placed anterior to the right side of the intraparietal bone. The screws were then attached to a headmount (Pinnacle Technology), which was fixed using dental cement. Following surgery, the animals were given 7 d to recover. Chronic 24-h EEG recordings were acquired using a 100× gain preamplifier high-pass filtered at 1.0 Hz (Pinnacle Technology) with video monitoring for two weeks. LabChart Pro software (ADInstruments, RRID:SCR_001620) was used for data acquisition and analysis.

### Preparation of acute brain slices

Cortical brain slices containing sensorimotor cortex (400 µM) were prepared from mice of either sex. Briefly, mice were anesthetized with isoflurane, decapitated, and the brains were rapidly removed and placed in chilled (4°C) low-Ca, low-Na slicing solution consisting of 234 mM sucrose, 11 mM glucose, 24 mM NaHCO_2_, 2.5 mM KCl, 1.25 mM NaH_2_PO_4_, 10 mM MgSO_4_, and 0.5 mM CaCl_2_, equilibrated with a mixture of 95% O_2_/5% CO_2_. The brain was glued to the slicing stage of a Vibratome 3000 sectioning system and slices were cut in a coronal orientation. The slices were then incubated in 32°C oxygenated aCSF (126 mM NaCl, 2.5 mM KCl, 1.25 mM NaH_2_PO_4_, 1 mM MgSO_4_, 2 mM CaCl_2_, 10 mM glucose, and 26 mM NaHCO_2_) for 1 h, and then allowed to cool to room temperature and subsequently used for recording.

### Field recordings

Slices were placed in an interface chamber maintained at 34°C, superfused with oxygenated aCSF at 2 ml/min and cortical projections were stimulated with a tungsten concentric bipolar electrode at the Layer VI, white matter boundary. Electrical stimulation consisted of 10–50 µA, 100-µs pulses at 30-s intervals delivered by a stimulus isolator (World Precision Instruments). Glass micropipettes (resistance ≅ 1 MΩ) were filled with aCSF and placed in Layer V ∼200 µm from the site of injury in the PMZ of FL animals (or comparable area of cortex in sham-injured animals) directly above the stimulation electrode. Electrophysiological data were recorded with an Axon Multiclamp 700A amplifier and Digidata 1322A digitizer (sampling rate = 20 kHz) with Lab Chart software (AD Instruments, RRID:SCR_001620). Threshold stimulation intensity was identified as the minimum amount of current required to elicit a detectable cortical field potential response (≥0.05 mV).

### Whole-cell patch-clamp recordings

Slices were placed in the recording chamber of an Olympus Bx51 microscope with continual superfusion of oxygenated aCSF maintained at 32°C (2 ml/min). Layer V pyramidal neurons were visually identified with infrared differential interference contrast microscopy and whole-cell patch-clamp recordings were made with a borosilicate glass electrode (3–5 MΩ) filled with 140 mM CsMs, 10 mM HEPES, 5 mM NaCl, 0.2 mM EGTA, 5 mM Qx314, 1.8 mM MgATP, and 0.3 mM NaGTP; pH 7.25. The recording electrode was placed ∼200 µm from the site of injury in the PMZ of FL animals, or comparable cortical area in sham-injured animals. Data were collected using an axon Multiclamp 700B amplifier, Digidata 1440A digitizer and pClamp software. Miniature EPSCs (mEPSCs) were recorded at a holding potential of −70mV in the presence of 1µM TTX. Only recordings with an access resistance that varied <20% were accepted for analysis.

### Electrophysiological data analysis

Field recordings were analyzed using Lab Chart (AD Instruments, RRID:SCR_001620), pClamp (Molecular Devices, RRID:SCR_011323) and MATLAB software (RRID:SCR_001622). Traces were recorded at threshold stimulation, the minimum stimulation required to elicit a detectable response. Each sweep was analyzed for epileptiform activity to calculate the percentage of epileptiform activity per slice. The area under the curve was used to determine the integrated network activity and was calculated by integrating the extracellular field potential during the first 1000 ms following initial stimulation. mEPSC recordings were analyzed using Clampfit (Molecular Devices, RRID:SCR_011323) and Mini Analysis (Synaptosoft, RRID:SCR_002184). Recordings of 60–120 s were analyzed for average amplitude and interevent frequency.

### Immunohistochemistry

Fixed mouse brains were prepared by transcardial perfusion with PBS followed by overnight fixation with 4% paraformaldehyde. Fixed brains were sectioned at 40 µm using a Thermo Fisher Microm HM 525 cryostat. Brain sections were blocked using blocking buffer (5% normal goat serum, 1% bovine serum albumin, in PBS) for 1 h at room temperature. Glial fibrillary acid protein (GFAP) (1:500, Abcam, RRID:AB_305808), NeuN (1:1000, Millipore, RRID:AB_2298772), CUX1 (1:100, Santa Cruz, RRID:AB_2261231), CTIP2 (1:500, Abcam, RRID:AB_2064130), and activated caspase-3 (1:500, Abcam, RRID:AB_443014) antibodies were diluted in PBS with 2% Triton X-100 and 5% blocking buffer. Cortical sections were incubated with diluted primary antibodies overnight at 4°C. Appropriate secondary antibodies (The Jackson Laboratory) were diluted in PBS with 5% blocking buffer and added to cortical sections for 2 h at room temperature. Slices were mounted using Vectashield (Vector Labs) and imaged with a Keyence BZ-X700 fluorescence microscope or Nikon A1R confocal microscope. All staining was analyzed with ImageJ (RRID:SCR_003070).

### Synapse counting

Tissue preparation, staining and analysis was adapted from the protocol from Ippolito and Eroglu (Ippolito and Eroglu, 2010). Brains were fixed as above and sectioned at 14 µm. Brain sections were blocked using 20% normal goat serum in PBS for 1 h at room temperature. Vglut1 (1:2500, Millipore, RRID:AB_2301751), PSD95 (1:500, Invitrogen, RRID:AB_2533914), and GFAP (1:1000, Abcam, RRID:AB_296804) were diluted in PBS with 0.3% Triton X-100 and 10% normal goat serum. Cortical sections were incubated with diluted primary antibodies for 42 h at 4°C. Appropriate secondary antibodies (The Jackson Laboratory) were diluted in PBS with 0.3% Triton X-100 and 10% normal goat serum and added to cortical sections for 2 h at room temperature. Slices were mounted using fluoromount-G (Southern Biotech) and imaged with a Nikon A1R confocal microscope. Images were collected with a 100× oil immersion objective and maximum intensity projections (MIPs), of three serial optical sections at 0.175-μm steps, were generated. Five MIPs were generated and analyzed per section. These were averaged to give one value per brain section. Synapses were quantified in ImageJ (RRID:SCR_003070) using the Puncta Analyzer plug in.

### Terminal deoxynucleotidyl transferase dUTP nick end labeling (TUNEL) assay

Brains were prepared by transcardial perfusion with PBS followed by overnight fixation with 4% paraformaldehyde. Fixed brains were sectioned at 40 µm using a Thermo Fisher Microm HM 525 cryostat. TUNEL was detected using an ApopTag peroxidase *in situ* apoptosis detection kit (Millipore). Briefly, endogenous peroxidase activity was blocked by incubation of the sections with 3% H_2_O_2_ for 5 min. TdT enzyme provided by the kit was used to label dUTP nick ends with DAB as the substrate. Nuclei were counterstained with methyl green and slices were mounted using Permount (Fisher Scientific). Tissue sections were evaluated by conventional bright-field microscopy and analyzed in ImageJ. The color deconvolution plug-in was used to isolate the DAB component of the staining. TUNEL-positive cells were quantified using the puncta analyzer in ImageJ. A uniform threshold and puncta size was applied across all images. The density of TUNEL-positive cells was quantified based on the number of TUNEL-positive cells in the region of interest which spanned from pial surface to the cortex white matter border and ∼500 μm on either side of the site of lesion.

### Experimental design and statistical analysis

For all studies, control experiments were performed including sham injury and age-matched vehicle treatment. Additionally, WT littermates were used as controls in experiments testing the effect of genetic deletion of α2δ-1. Multiple litters were used for each experiment to control for litter to litter differences. GBP versus vehicle and sham versus FL was randomized by litter, ensuring a mix of treatments and genotypes throughout all studies. The distribution of genotypes followed a Mendelian distribution. The sex of the mice was ∼50% male and 50% female. Animals were randomly assigned to one of four treatment groups. The experimenter was blind to genotype at the time of FL and drug treatment. Data analysis was not blinded, but many assays use automated, quantitative analysis which helps to reduce potential bias in many experiments. The majority of experiments in this study had three between-subject factors being tested: sham versus FL, WT versus α2δ-1 KO, and vehicle versus GBP treatment. Each experimental group for electrophysiology and immunohistochemistry experiments consisted of three to four animals, with eight different experimental groups (WT sham +vehicle, KO sham +vehicle, WT sham +GBP, KO sham +GBP, WT FL +vehicle, KO FL +vehicle, WT FL +GBP, and KO WT +GBP). Exact animal number and slice/cell number is noted in the results. Sample size has been calculated to ensure power >0.8 for α = 0.05. Power was calculated using G*Power Software. All experiments used animals of the same age between groups.

For comparison between two experimental groups, a Student’s *t* test was used. To control for multiple comparisons (with three or more experimental groups), a one-way ANOVA was used. For comparison of cumulative probabilities, a Kolmogorov-Smirnov test (KS test) was used. For all experiments, we compared WT FL to WT Sham, WT FL to WT FL + GBP, WT FL to KO FL, and KO FL to KO FL + GBP. To control for multiple comparisons, a Holm-Bonferroni correction was used. All data are presented as box-whisker plots ± SEM, with average values indicated by X on the plots. Exact P values are indicated in the results, unless they were <0.001; values of *p* < 0.05 for α = 0.05 or less were considered statistically significant. OriginLab and MATLAB software was used to perform the statistical analyses.

In addition, to perform more rigorous statistical analysis linear mixed-effects models were fitted with random intercepts to account for the correlation between repeated measurements on the same mouse. To standardize the modeling procedure and facilitate comparison across the experiments, fixed effects always included main effects for FL, drug treatment, and genetic KO of α2δ-1, as well as interaction terms between FL and drug treatment, FL and genetic KO of α2δ-1, and drug treatment and genetic KO of α2δ-1. In other words, sham, vehicle, and WT served as reference categories. *t* values > 1.96 and < −1.96 we considered to be statistically significant.

### Drugs and reagents

All salts and glucose for buffers were obtained from Sigma-Aldrich. GBP was obtained from Abcam.

## Results

### Pharmacological attenuation of α2δ-1 signaling decreases cell death following FL

**Figure 1.**
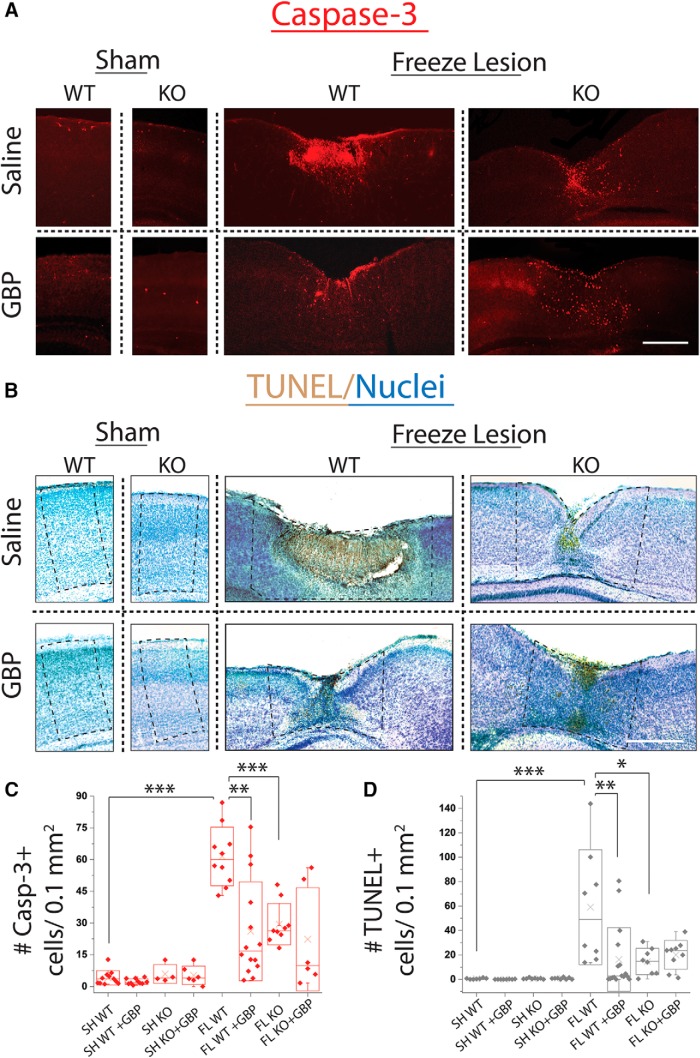
GBP treatment or α2δ-1 deletion decreases cell death following FL. ***A***, Representative images of cleaved caspase-3 (casp-3) staining in P7 WT and α2δ-1^−/−^ sham ±GBP and WT and α2δ-1^−/−^ FL ±GBP. Scale bar = 500 µm. ***B***, Representative bright-field images of TUNEL assay (TUNEL+ cells stained brown by DAB, nuclei counterstained by methyl green) in P7 WT and α2δ-1^−/−^(KO) sham ±GBP and WT and α2δ-1^−/−^ FL ±GBP. Scale bar = 500 µm. Approximate ROI for analysis shown in dashed box. ***C***, Box-whisker plot of casp-3+ cells per 0.1 mm^2^, **α = 0.01 and ***α = 0.001 (Holm-Bonferroni multiple-comparison correction). ***D***, Box-whisker plot of TUNEL+ cells per section, *α = 0.05, **α = 0.01, and ***α = 0.001 (Holm-Bonferroni multiple-comparison correction).

GBP treatment has been shown to reduce cell death in models of posttraumatic epilepsy (Li et al., 2012) and stroke (Traa et al., 2008), suggesting that α2δ-1 signaling may contribute to insult-induced cortical cell death. Here, we tested whether attenuating α2δ-1 signaling via *in vivo* GBP treatment was neuroprotective in the FL model. As described in previous studies, FL was performed in C57BL/6J mice on the day of birth (P0). To identify dead and dying cells in the neonatal cortex, we used TUNEL labeling and activated caspase-3 immunolabeling (Watanabe et al., 2002). We found that FL induced significant cell death in the cortex at P7 (Fig. 1; caspase-3 immunoreactivity: sham, 4.1 ± 1.0 cells per 0.1 mm^2^, *n* = 11 sections from four animals; FL, 61.5 ± 4.4 cells per 0.1 mm^2^, *n* = 10 sections from three animals; Mann-Whitney, *U*_(19)_ = 0, *p* < 0.001; Holm-Bonferroni correction for multiple comparisons, α = 0.001; TUNEL: sham, 0.5 ± 0.2 cells per 0.1 mm^2^, *n* = 7 sections from three animals; FL, 59.1 ± 16.6 cells per 0.1 mm^2^, *n* = 8 sections from four animals; Mann-Whitney, *U*_(13)_ = 0, *p* < 0.001; Holm-Bonferroni correction for multiple comparisons, α = 0.001). The majority of cell death was seen at the site of FL and in the surrounding cortical tissue. Other developmental time points were examined and the peak expression of cell death markers was seen at approximately P5–P7 (data not shown). Therefore, the effects of α2δ-1 signaling on cell death were quantified at P7.

To determine the role of α2δ-1 signaling in FL-induced cell death, we first treated animals with GBP after FL. Sham and FL animals were treated with 200 mg/kg GBP or vehicle (intraperitoneal injection) once daily from P1 to P7. We found that GBP treatment significantly reduced cell death following FL, as measured using both assays (Fig. 1; caspase-3 immunoreactivity: 26.1 ± 6.2 cells per 0.1 mm^2^, *n* = 14 sections from four animals; Mann-Whitney, *U*_(22)_ = 122, *p* = 0.003; Holm-Bonferroni correction for multiple comparisons, α = 0.01; TUNEL: 19.9 ± 7.7 cells per 0.1 mm^2^, *n* = 13 sections from four animals; *U*_(19)_ = 219, *p* < 0.001; Holm-Bonferroni correction for multiple comparisons, α = 0.001). Very few cells expressed either marker of cell death in GBP-treated sham animals (caspase-3 immunoreactivity: 2.3 ± 0.4 cells per 0.1 mm^2^, *n* = 10 sections from three animals; TUNEL: 0.1 ± 0.04 cells per 0.1 mm^2^, *n* = 8 sections from three animals).

### Genetic deletion of α2δ-1 attenuates FL-induced cell death

Next, we examined whether genetic deletion of α2δ-1 recapitulated GBP’s neuroprotective effects after FL. Mice lacking the α2δ-1 gene *CACNA2D1* (α2δ-1 KOs) were generated and the absence of the α2δ-1 protein was confirmed by Western blot analysis of total cortical homogenate (data not shown). These mice showed no obvious phenotype and developed normally with regard to size and weight. α2δ-1 KO mice were recently reported to have a higher risk of developing diabetes due to changes in pancreatic β-cell function (Mastrolia et al., 2017). α2δ-1 KOs showed no change in open field activity or time spent in the center of the open field and performed normally on the rotarod and alternating T-maze test (data not shown). No behavioral or electrographic seizures were detected in α2δ-1 KOs subjected to two weeks of 24/7 video/EEG monitoring (data not shown).

After characterizing the α2δ-1 KO mice, we performed FL experiments. We found that mice lacking α2δ-1 (α2δ-1 KO) had reduced cell death at P7 following FL (Fig. 1; caspase-3 immunoreactivity: 38.8 ± 9.7 cells per 0.1 mm^2^, *n* = 10 sections from three animals; *t* test, *t*_(17)_ = 5.75, *p* < 0.001 as compared to WT FL; Holm-Bonferroni correction for multiple comparisons, α = 0.001; TUNEL: 14.6 ± 3.8 cells per 0.1 mm^2^, *n* = 7 sections from three animals; *t* test, *t*_(14)_ = 2.79, *p* = 0.01; Holm-Bonferroni correction for multiple comparisons, α = 0.05), consistent with α2δ-1 signaling contributing to FL-induced cell death. Previously, it has been unknown whether GBP’s effects on cell death act via α2δ-1 signaling or via another pathway. To test this, we treated α2δ-1 KO animals with GBP after FL. GBP treatment did not further reduce cell death in α2δ-1 KO animals (Fig. 1; caspase-3 immunoreactivity: 22.3 ± 9.9 cells per 0.1 mm^2^, *n* = 6 sections from three animals; TUNEL: 19.7 ± 3.4 cells per 0.1 mm^2^, *n* = 8 sections from three animals). This indicates that GBP likely acts via α2δ-1 to reduce cell death, as GBP has no additional neuroprotective effects in α2δ-1 KO mice. Neither GBP treatment, nor genetic deletion of α2δ-1, completely eliminated cell death consistent with other cell death pathways likely contributing to neuronal loss following FL.

### Attenuating α2δ-1 signaling reduces FL-induced cortical malformation

**Figure 2.**
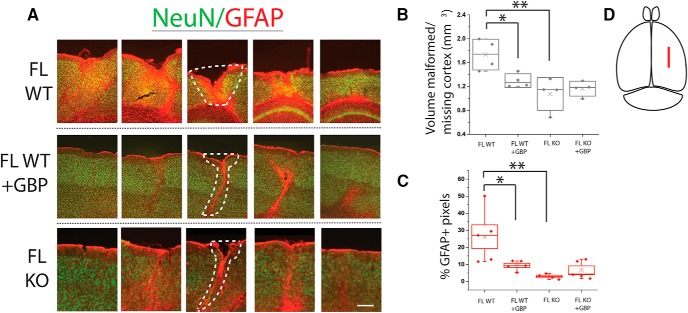
GBP treatment or α2δ-1 KO decreases malformation volume and astrocyte reactivity after FL. ***A***, GFAP (red) and NeuN (green) staining in P28 WT FL, WT GBP-treated FL, and α2δ-1^−/−^ (KO) FL cortex. Serial sections taken at 480-µm intervals, spanning the total length of the MZ. Scale bar = 100 µm. Dotted white line showing area of both missing and malformed cortical tissue. ***B***, Box-whisker plot of volume of missing or malformed cortex (mm^3^), estimated from area calculated in serial sections, *p < 0.05 and **p < 0.01. ***C***, Box-whisker plot of percentage of GFAP-positive pixels in ∼600 µm^2^ ROI from white matter to the pial surface, centered around the MZ, *p < 0.05 and **p < 0.01. ***D***, Illustration depicting location of FL injury.

Because genetic and pharmacological inhibition of α2δ-1 signaling reduced cell death, we hypothesized that similar manipulations would decrease FL-induced cortical reorganization. To test this hypothesis, we examined anatomic changes induced by FL in GBP-treated and α2δ-1 KO animals. Because FL creates a 3-dimensional cortical malformation, we prepared serial cortical brain sections from P28 FL animals that encompassed the entire FL (Fig. 2*D*). We identified areas of malformation, characterized by large-scale cortical disorganization, by examining NeuN (marker of neurons) immunolabeled sections. The area of malformed, or missing, cortex was quantified throughout the reconstructed cortex. When FL resulted in loss of cortical tissue, this loss was quantified by drawing a line continuous with the adjacent pial surface and calculating the area of predicted tissue loss. The area of missing cortex was added to the area of malformed cortex to calculate lesion area per slice. The area was calculated in serial sections through the volume of the lesion and used to estimate the total lesion volume. Consistent with our cell death data, the volume of the lesion was decreased in FL animals treated with GBP from P1 to P7 (Fig. 2*A*,*B*; FL: 1.65 ± 0.13 mm^3^, *n* = three animals vs FL + GBP: 1.25 ± 0.03, *n* = three animals; one-way ANOVA, *F*_(3)_ = 8.01, *p* = 0.003; *post hoc* Bonferroni test, α = 0.05). α2δ-1 KO mice also showed reduced lesion size compared to WT FL (Fig. 2*A*,*B*; 1.08 ± 0.13 mm^3^, *n* = 4 and 1.16 ± 0.06 mm^3^, *n* = 4 for vehicle and GBP-treated animals, respectively; *post hoc* Bonferroni test, α = 0.05). These results demonstrate that reducing α2δ-1 signaling with either GBP treatment or α2δ-1 KO reduces the areas of cortical malformation following FL. Similar to cell death data, treating α2δ-1 KO animals with GBP did not result in additional reductions in cortical malformation (Fig. 2*A*,*B*; 1.16 ± 0.06 mm^3^, *n* = 4). This again suggests that GBP’s neuroprotective effects are mediated by α2δ-1.

### Attenuating α2δ-1 signaling decreases GFAP immunoreactivity following FL

In parallel to measuring lesion volume, we also examined immunoreactivity for GFAP, a protein that is strongly upregulated by reactive astrocytes in the cerebral cortex. Our previous work, and the work of others, shows that FL causes significant reactive astrocytosis (Bordey et al., 2001; Dulla et al., 2013; Armbruster et al., 2014; Campbell et al., 2014; Hanson et al., 2016). To quantify GFAP labeling in the cortex, we examined immunolabeled serial sections through the entire FL volume at P28. GBP treatment significantly reduced FL-induced increases in GFAP immunolabeling (Fig. 2*A*,*D*; 9.47% ± 1.24 GFAP+ pixels, *n* = 5 animals) compared to vehicle treated FL animals (26.23% ± 6.97, *n* = 5 animals; one-way ANOVA, *F*_(3)_ = 6.73, *p* = 0.004; *post hoc* Bonferroni test, α = 0.05). Similarly, genetic deletion of α2δ-1 KO reduced GFAP immunolabeling after FL (Fig. 2*A*,*D*; 2.92% ± 0.74, *n* = 4; *post hoc* Bonferroni test, α = 0.01) without any additive effects of GBP treatment (6.77% ± 2.36, *n* = 4). Consistent with previous findings, these results show that GBP treatment reduces astrocyte reactivity via its actions on α2δ-1.

### FL induces α2δ-1-dependent laminar reorganization of the cortex

**Figure 3.**
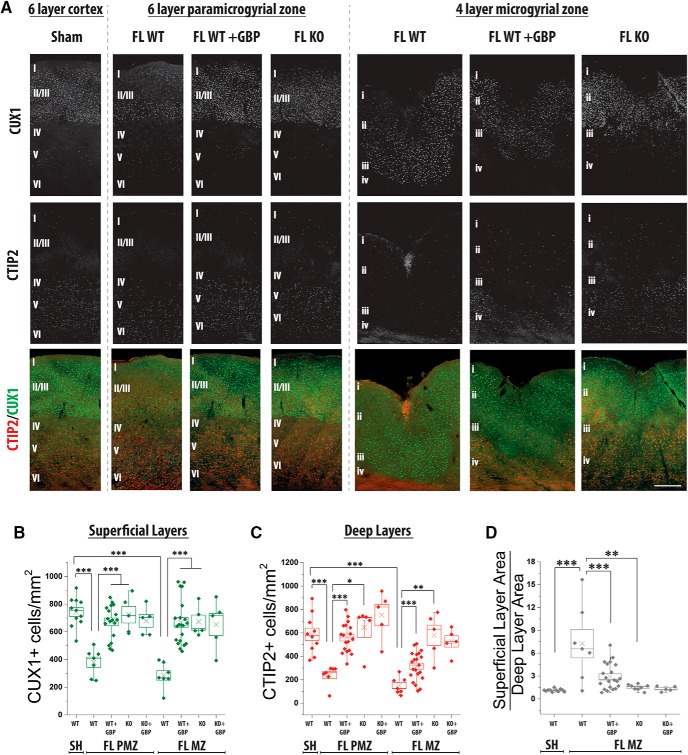
GBP treatment or α2δ-1 KO attenuates the loss of layer-specific markers in the FL cortex. ***A***, Representative images of CUX1 (green) and CTIP2 (red) staining in P28 WT sham cortex and the PMZ and MZ of WT FL, WT FL + GBP, and α2δ-1^−/−^ (KO) FL cortex. Scale bar = 250 µm. ***B***, Box-whisker plot of CUX1+ cells per mm^2^ in the outer layers, ***α = 0.001 (Holm-Bonferroni multiple-comparison correction). ***C***, Box-whisker plot of CTIP2+ cells per mm^2^ in the deep layers, ***α = 0.001, **α = 0.01, and *α = 0.05 (Holm-Bonferroni multiple-comparison correction). ***D***, Box-whisker plot of ratio of area of outer layer (Layer II/III in sham and layer ii in FL) to area of inner layer (Layer V and VI in sham and layer iv in FL) in sham, FL, FL + GBP, and α2δ-1 KO FL cortex, ***α = 0.001 and **α = 0.01 (Holm-Bonferroni multiple-comparison correction).

In order to better understand FL-induced anatomic changes, we examined the expression of layer specific markers in the cortex at P28. CUX1 and CTIP2 are transcription factors that preferentially label superficial (LII/III) and deep (LIV/VI) cortical layers, respectively (Insolera et al., 2014). Consistent with published data, CTIP2+ cells are abundant in deep cortical layers (Fig. 3*A*; 588 ± 52 per mm^2^, *n* = 11 sections from four animals), and found sporadically in outer layers (Fig. 3*A*; 26 ± 5 per mm^2^) in the sham-injured cortex. Conversely, CUX1+ cells are more abundant in the outer layers and are sparser in deep layers (Fig. 3*A*; 743 ± 31 vs 119 ± 26 per mm^2^). In contrast to the normal six-layered cortex, FL creates a four-layered MZ. Layer i is continuous with cortical Layer I (lower case = FL nomenclature, upper case = standard cortical layer nomenclature), and is the superficial most layer. It is thin and contains few cell bodies. Layer ii is neuron-rich and makes up the bulk of the MZ (Dvorák and Feit, 1977) and contains many cell bodies. Layer iii is a neuron-free area, and makes a clear border between superficial and deep cortical layers. Layer iv is identified as the deepest layer of cell bodies above the white matter. We defined layer i and ii as the superficial layers of the MZ, and layer iv as the deep layer. Studies postulate that neurons in MZ layer ii are similar to Layer II/III neurons and that Layer IV-VI cortical neurons are lost, but the expression of layer-specific markers following FL has not been examined. We found that layer ii of the MZ is predominately composed of CUX1+ cells and layer iv of CTIP2+ cells, in line with previous predictions. Interestingly, there is a decreased density of CUX1+ cells in MZ layer ii (Fig. 3*A*,*B*; 285 ± 34 per mm^2^, *n* = 7 sections from three animals; *t* test, *t*_(16)_ = 9.40, *p* < 0.001, compared to sham-injured Layer II/III; Holm-Bonferroni correction for multiple comparisons, α = 0.001) and CTIP2+ cells in MZ layer iv (Fig. 3*A*,*C*; 240 ± 31 per mm^2^; *t* test, *t*_(15)_ = 6.47, *p* < 0.001, compared to Layer V–VI; Holm-Bonferroni correction for multiple comparisons, α = 0.001). Additionally, the ratio of superficial layer (layer ii or II/III) to deep layer (iv or VVI) is significantly higher in FL MZ cortex (Fig. 3*D*; 7.26 ± 1.87, *n* = 7 sections from three animals) compared to sham-injured cortex (1.12 ± 0.06, *n* = 11 sections from four animals; *t* test, *t*_(16)_ = −4.17, *p* < 0.001; Holm-Bonferroni correction for multiple comparisons, α = 0.001) reflecting the loss of deep layer cells and concurrent expansion of the outer layer in FL cortex.

Based on these findings, we next examined how attenuating α2δ-1 signaling affects layer-specific changes in the MZ. Sham and FL animals were treated with GBP, as described above, and CTIP2+ and CUX1+ cells were identified at P28. GBP treatment restored the density of CUX1+ cells in the outer layers to sham-injured levels (Fig. 3*A*,*B*; 668 ± 32 per mm^2^, *n* = 21 sections from four animals; Mann-Whitney, *U*_(23)_ = 0, *p* < 0.001, compared to vehicle-treated FL; Holm-Bonferroni correction for multiple comparisons, α = 0.001) and significantly increased the density of deep layer CTIP2+ cells (Fig. 3*A*,*C*; 111 ± 14 per mm^2^; *t* test, *t*_(26)_ = −3.43, *p* < 0.001, compared to vehicle-treated FL; Holm-Bonferroni correction for multiple comparisons, α = 0.001). Consistent with a significant reduction of lesion volume, FL animals treated with GBP have a significantly smaller superficial/deep layer ratio of 2.93 ± 0.37 (Fig. 3*D*; *n* = 21 sections from four animals; *t* test, *t*_(26)_ = 3.53, *p* < 0.001; Holm-Bonferroni correction for multiple comparisons, α = 0.001) and the MZ more closely resembles the normal six-layered cortex.

We next examined how genetic deletion of α2δ-1 affected laminar reorganization in the MZ. α2δ-1 KO attenuated FL-induced decreases in CUX1+ and CTIP2+ cell density (Fig. 3*A–C*; 723 ± 59, *n* = 5 sections from three animals; *t* test, *t*_(10)_ = −6.68, *p* < 0.001; Holm-Bonferroni correction for multiple comparisons, α = 0.001 and 646 ± 84 cells per mm^2^; *t* test, *t*_(8)_ = −6.68, *p* < 0.001, compared to WT FL; Holm-Bonferroni correction for multiple comparisons, α = 0.01). FL-induced changes in the ratio of superficial/deep layers were also significantly attenuated in α2δ-1 KO animals (Fig. 3*D*; 1.52 ± 0.13, *n* = 8 sections from three animals; *t* test, *t*_(17)_ = −3.00, *p* < 0.001; Holm-Bonferroni correction for multiple comparisons, α = 0.01). GBP-treatment did not affect CUX1+ and CTIP2+ cell density in α2δ-1 KO mice (Fig. 3*A–C*; 692 ± 81 and 676 ± 103 cells per mm^2^, *n* = 7 sections from three animals).

We next examined the cellular density of CUX1 and CTIP2 in the PMZ, the area immediately adjacent to the MZ, which is responsible for generating epileptiform network activity. Interestingly, the PMZ has long been considered an area of normal cortical lamination. While gross cortical lamination is normal, we found that FL alters the density of CUX1+ and CTIP2+ cells in the PMZ. The density of CUX1+ cells in superficial layers (Fig. 3*A*,*B*; 380 ± 37 cells per mm^2^; *t* test, *t*_(16)_ = 9.40, *p* < 0.001; Holm-Bonferroni correction for multiple comparisons, α = 0.001) and density of CTIP2+ cells in deep layers (Fig. 3*A*,*C*; 240 ± 31 cells per mm^2^; Mann-Whitney, *U*_(16)_ = 70, *p* = 0.0001; Holm-Bonferroni correction for multiple comparisons, α = 0.001) were significantly reduced in the PMZ as compared to sham-injured cortex. GBP treatment prevented the layer-specific decreases in CUX1+ and CTIP2+ cells in the PMZ (671 ± 28; Mann-Whitney, *U*_(23)_ = 0, *p* < 0.001; Holm-Bonferroni correction for multiple comparisons, α = 0.001 and 560 ± 29 cells per mm^2^; Mann-Whitney, *U*_(23)_ = 0, *p* < 0.001; Holm-Bonferroni correction for multiple comparisons, α = 0.001, respectively), as does α2δ-1 KO (672 ± 48; *t* test, *t*_(10)_ = −5.16, *p* = 0.0004 and 579 ± 86 cells per mm^2^; Mann-Whitney, *U*_(10)_ = 0, *p* = 0.006; Holm-Bonferroni correction for multiple comparisons, α = 0.05). GBP treatment did not provide further protection against anatomic reorganization, again suggesting GBP mediates it protective effects via α2δ-1. These results demonstrate that FL disrupts cortical lamination via α2δ-1 signaling, as assayed by the expression of layer-specific markers.

### Total neuron number is normal in the FL cortex

Because the expression of CUX1 and CTIP2 was altered by FL, we wondered whether neuronal density was also affected. To test this, we performed NeuN staining and counted the total number of neurons in the cortex. Confocal images of the outer and deep cortical layer were used to determine neuronal density, in sham-injured cortex and in the MZ and PMZ of the FL cortex. In comparison to the dramatic changes found in CUX1 and CTIP2 staining following FL, no significant reduction in total neuron number was observed (data not shown). These results suggest that overall neuronal density is normal in the FL cortex at P28, despite changes in CUX1+ and CTIP2+ cell density.

### Decreasing α2δ-1 signaling attenuates FL-driven increases in synaptogenesis

**Figure 4.**
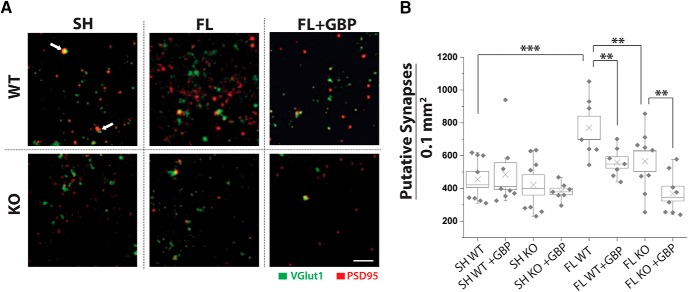
GBP treatment or α2δ-1 deletion decreases FL-driven synaptogenesis. ***A***, MIPs from of three optical sections of confocal images of VGlut1 (green) and PSD95 (red) collected at 100× from Layer V of WT and α2δ-1^−/−^ (KO) FL and sham-injured animals, with vehicle or GBP treatment. White arrow indicates colocalization of VGLUT1 and PSD95, representing a site of synaptic contact. Scale bar = 500 nm. ***B***, Box-whisker plot of number of synapses (colocalization of VGlut1/PSD95) per 1.00 mm^3^ per MIP, **α = 0.01 and ***α = 0.001 (Holm-Bonferroni multiple-comparison correction).

GBP decreases excitatory synaptogenesis during development (Eroglu et al., 2009) and following injury (Li et al., 2012; Li et al., 2014). We have previously shown that GBP treatment can block FL-induced cortical network hyperexcitability and increased EPSC frequency (Andresen et al., 2014). We hypothesized that hyperexcitability was prevented by attenuating α2δ-1-driven aberrant excitatory synaptogenesis in the FL cortex. Here, we examined excitatory synapse number by immunolabeling with the pre- and postsynaptic markers, VGLUT1 and PSD95. Synapse counting was performed at P28 in Layer V adjacent to the injury (PMZ), as this is the region that was previously shown to be hyperexcitable and to have increased excitatory synaptic activity following FL. Colocalization of VGLUT1 and PSD95 puncta was quantified (Ippolito and Eroglu, 2010) to determine the density of putative excitatory synaptic contacts. We found that sham-injured WT animals had a synaptic density of 488 ± 70 per 0.1 mm^2^ (*n* = 8 sections from three animals; Fig. 4). FL induced a marked increase in the density of putative excitatory synapses in WT mice (Fig. 4*A*,*B*; 758 ± 68 per 0.1 mm^2^, *n* = 7 sections from three animals; Mann-Whitney, *U*_(13)_ = 3, *p* < 0.001 compared to WT sham; Holm-Bonferroni correction for multiple comparisons, α = 0.001). Both GBP treatment and genetic deletion of α2δ-1 attenuated FL-induced increase in colocalized VGLUT1/PSD95 puncta (Fig. 4*A*,*B*; 567 ± 41, *n* = 7 sections from three animals; *t* test, *t*_(16)_ = 3.50, *p* = 0.0029; Holm-Bonferroni correction for multiple comparisons, α = 0.01 and 556 ± 60, *n* = 9 sections from three animals; *t* test, *t*_(22)_ = 3.55, *p* = 0.0018 compared to WT FL; Holm-Bonferroni correction for multiple comparisons, α = 0.01). In contrast to our other results, GBP treatment further reduced synapse number in the FL α2δ-1 KO mice (368 ± 45 per 0.1 mm^2^, *n* = 8 sections from three animals, *t* test, *t*_(19)_ = 3.28, *p* = 0.004; Holm-Bonferroni correction for multiple comparisons, α = 0.01). These results demonstrate that FL induces α2δ-1-mediated synaptogenesis, but that GBP also has α2δ-1-independent effects on synaptogenesis.

### Attenuating α2δ-1 signaling inhibits FL-driven increases in synaptic excitation

**Figure 5.**
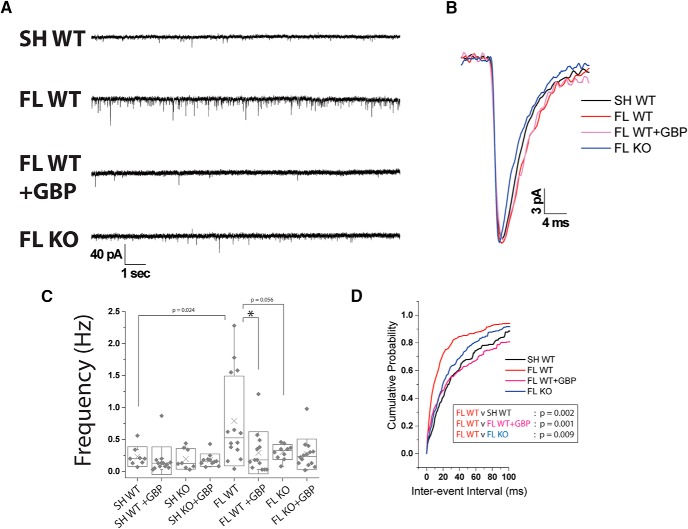
GBP treatment or α2δ-1 KO attenuates the rise in mEPSC frequency in the FL cortex. ***A***, Representative miniature excitatory postsynaptic current recording of Layer V pyramidal neuron recorded from the PMZ of acute cortical slices prepared from P21 to P28 WT sham, WT FL, WT FL + GBP, and α2δ-1^−/−^ (KO) FL. ***B***, Example mEPSC from WT sham (black), WT FL (red), WT FL + GBP (pink), and α2δ-1^−/−^ FL (blue). ***C***, Box-whisker plot of frequency of mEPSC events, *α = 0.05 (Holm-Bonferroni multiple-comparison correction). ***D***, Cumulative probability of IEIs of WT sham (black), WT FL (red), WT FL + GBP (pink), and α2δ-1^−/−^ FL (blue). WT FL has a significant rightward shift compared to WT sham, WT FL + GBP, and α2δ-1^-/-^ FL, *p* < 0.001 (KS test).

Given the changes in excitatory synapse number, we next assessed the role of α2δ-1 signaling on synaptic excitation by examining mEPSCs in the FL cortex. mEPSCs were recorded from Layer V pyramidal neurons ∼200 µm from the site of injury in the PMZ of FL animals, or comparable cortical area in sham-injured animals. Neurons were held at -70 mV and perfused with normal aCSF containing 1 μm TTX. We found no significant differences in resting membrane potential, rise or decay kinetics, or the amplitude of the mEPSC events between groups (Fig. 5*B*). Similar to our previous study (Andresen et al., 2014), we found that FL induced a rise in mEPSC frequency (Fig. 5*A*,*C*; 0.79 ± 0.19 Hz, *n* = 14 cells from three animals; Mann-Whitney, *U*_(20)_ = 22.5, *p* = 0.002; Holm-Bonferroni correction for multiple comparisons, α = 0.05) compared to sham-injured animals (0.23 ± 0.05 Hz, *n* = 8 cells from three animals). GBP treatment significantly reduced the mEPSC frequency (0.29 ± 0.09 Hz, *n* = 13 cells from three animals; Mann-Whitney, *U*_(25)_ = 142.5, *p* = 0.013; Holm-Bonferroni correction for multiple comparisons, α = 0.05). α2δ-1 deletion led to a non-significant reduction in mean mEPSC frequency (0.30 ± 0.04 Hz, *n* = 10 cells from three animals; Mann-Whitney, *U*_(22)_ = 103, *p* = 0.055) following FL. GBP treatment had no effect on mEPSC frequency in α2δ-1 KO mice. The cumulative probability distribution of the interevent interval (IEI) was also calculated to more rigorously assess changes in mEPSC frequency. The leftward shift in the IEI cumulative probability in WT FL cortex, compared to WT sham, shows an increased frequency of excitatory synaptic events following FL (Fig. 5*D*; KS test, *D*_(21)_ = 0.71, *p* = 0.0002; Holm-Bonferroni correction for multiple comparisons, α = 0.001. The IEI cumulative probability is shifted rightward in both GBP treated WT FL and α2δ-1 KO FL animals (KS test, *D*_(26)_ = 0.82, *p* = 0.0001 and KS test, *D*_(22)_ = 0.64, *p* = 0.0009 compared to WT FL; Holm-Bonferroni correction for multiple comparisons, α = 0.001), demonstrating that both GBP treatment and α2δ-1 deletion reduces synaptic excitation.

### Genetic deletion of α2δ-1 attenuates FL-driven network hyperexcitability

**Figure 6.**
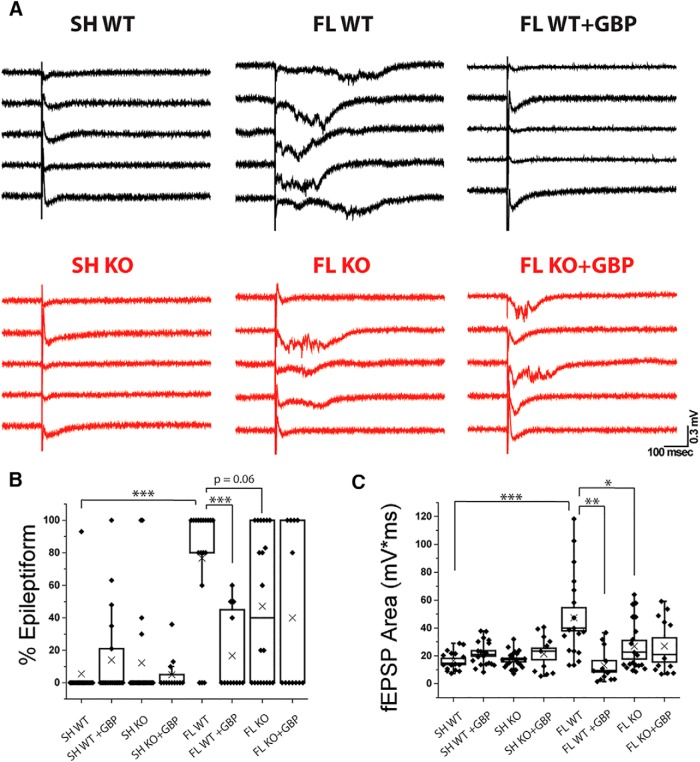
α2δ-1 KO partially rescues network hyperexcitability following FL and is insensitive to GBP treatment. ***A***, Representative evoked cortical field EPSP traces recorded from the PMZ of acute cortical slices prepared from P21 to P28 WT sham, α2δ-1^−/−^ sham, WT FL, α2δ-1^−/−^ FL, WT FL + GBP, and α2δ-1^−/−^ FL + GBP animals. ***B***, Box-whisker plot of percentage epileptiform sweeps per slice, ***α = 0.001 (Holm-Bonferroni multiple-comparison correction). ***C***, Box-whisker plot of integrated area under the curve per slice, *α = 0.05, **α = 0.01, and *α = 0.05 (Holm-Bonferroni multiple-comparison correction).

Epileptiform activity can be readily evoked in the adult FL cortex (Jacobs et al., 1996; Luhmann et al., 1996; Jacobs et al., 1999). In a previous study, we showed that GBP treatment prevents the development of evoked epileptiform field EPSCs (fEPSPs) recorded from acute cortical slices (Andresen et al., 2014). Given that α2δ-1 deletion prevents FL-induced excitatory synaptogenesis, we hypothesized that α2δ-1 KO would also decrease network hyperexcitability. To test this hypothesis, fEPSPs were evoked by stimulating the white matter beneath the cortex and recording from Layer V of the PMZ. Stimulation intensity was set as the minimum stimulation required to elicit a detectable response, and did not differ significantly between groups. We quantified the number of sweeps per slice with epileptiform activity (defined by high frequency activity, as well as increased amplitude and duration) and the integrated area under the curve of the fEPSP response. Slices from WT sham-injured animals show almost no epileptiform activity (Fig. 6*B*; 1%, *n* = 16 sections from four animals) and a small fEPSP area (Fig. 6*C*; 14.48 ± 1.75 mV*ms). Similar to previous studies, we found that FL animals have robust epileptiform activity (Fig. 6*A*,*B*; 76.6 ± 9%, *n* = 18 sections from four animals, Mann-Whitney, *U*_(33)_ = 32, *p* < 0.001; Holm-Bonferroni correction for multiple comparisons, α = 0.001), corresponding to a significantly larger fEPSP area (Fig. 6*C*; 47.35 ± 7.33 mV*ms, Mann-Whitney, *U*_(33)_ = 37, *p* < 0.001; Holm-Bonferroni correction for multiple comparisons, α = 0.001). GBP treatment significantly decreased the percentage epileptiform activity (Fig. 6*A*,*B*; 16.6 ± 7%, *n* = 12 section from three animals; Mann-Whitney, *U*_(28)_ = 191.5, *p* < 0.001; Holm-Bonferroni correction for multiple comparisons, α = 0.001) and fEPSP area (Fig. 6*C*; 13.02 ± 3.59 mV*ms; Mann-Whitney, *U*_(28)_ = 193, *p* < 0.001; Holm-Bonferroni correction for multiple comparisons, *p* < 0.01) following FL. Genetic deletion of α2δ-1 caused a non-significant trend toward decreased epileptiform activity following FL (Fig. 6*A*,*B*; 47.1 ± 10%, *n* = 20 sections from three animals; Mann-Whitney, *U*_(36)_ = 240.5, *p* = 0.06), and significantly decreased fEPSP area (Fig. 6*C*; 26.92 ± 4.14 mV*ms; Mann-Whitney, *U*_(36)_ = 262, *p* = 0.017; Holm-Bonferroni correction for multiple comparisons, α = 0.05) compared to WT FL. GBP treatment had no additive effect in α2δ-1 KO animals after FL on percentage epileptiform activity (Fig. 6*A*,*B*; 40.0 ± 14.3%, *n* = 12 sections from three animals) or fEPSP area (Fig. 6*C*; 26.95 ± 5.95 mV*ms) compared to vehicle-treated FL α2δ-1 KOs suggesting that the actions of GBP on network hyperexcitability are specific to antagonizing α2δ-1 signaling. These results suggest an intermediate effect of α2δ-1 deletion compared to acute pharmacological attenuation of α2δ-1 on network hyperexcitability.

### Linear mixed effects modeling of data to increase statistical rigor

Based on the call to increase rigor and reproducibility in preclinical studies, we performed a more advanced statistical analysis of all our results. The goal of this secondary level of analysis was to identify results that may require further studies to ensure their robustness and reproducibility. We employed a linear mixed effects model (Laird and Ware, 1982) that (1) includes mouse-specific random intercepts to account for correlation due to multiple measures in the same mouse, (2) allows us to look at the interactions between manipulations (FL, drug treatment, and genetic manipulation), and (3) encompasses the entire study design within a single statistical approach. For all studies described above, regression coefficients from the linear mixed-effects models were calculated (Table 1) and indicate the degree to which different experimental conditions alter the mean outcomes for each assay. The GBP main effect, for example, measures the change in mean outcome after GBP treatment specifically in sham-injured WT mice, though the effect also applies to all experimental groups that received GBP. The FL/GBP interaction term, on the other hand, measures the additional difference in the effect of GBP in FL versus sham-injured mice. Results varied by experiment, but generally agree with statistical analysis performed using Student’s *t* test/Mann-Whitney tests with a Bonferroni correction for repeated measures. The most robust effects based on linear mixed effects modeling were the (1) FL main effect, and the interactions among (2) FL/GBP, (3) FL/KO, and (4) GBP/KO. This indicates that (1) FL has robust effect across assays, (2/3) GBP and α2δ-1 KO have important effects in FL animals, and that (4) the effects of GBP are largely lost in α2δ-1 KO mice. Importantly, there are some results that have less statistical significance based on the linear mixed effects model. Less robust results include the effects of α2δ-1 KO on circuit hyperexcitability after FL, the effects of GBP treatment on the recovery of CTIP2, and the effects of α2δ-1 KO on the recovery of CUX1. These specific findings may warrant further study to ensure their robustness before moving forward with any clinical applications. In general, this additional statistical analysis shows that GBP and α2δ-1 have robust effects across multiple FL-associated pathologies.

**Table 1.** Linear mixed effects modeling table of coefficient, SE, and *t* value calculated from the linear mixed effect model for data from: TUNEL assay (Fig. 1), caspase-3 staining (Fig. 1), CUX1 and CTIP2 staining (Fig. 3), superficial/deep layer area ratio (Fig. 3), synapse counting (Fig. 4), fEPSP %epileptiform (Fig. 6), and fEPSP area (Fig. 6)

Linear mixed model fit									
Fixed effect	Coefficient	SE	*t* value	Significant	Fixed effect	Coefficient	SE	*t* value	Significant
TUNEL					Caspase-3				
FL Injury	4.88E-02	1.29E-02	3.798	*	FL Injury	5.17E-03	7.52E-04	6.870	*
GBP Treatment	−1.14E-02	1.31E-02	−0.871		GBP treatment	-7.84E-04	7.69E-04	-1.019	
α2δ-1 KO	−1.09E-02	1.31E-02	−0.831		α2δ-1 KO	−6.09E-04	8.46E-04	−0.720	
Interaction GBP/FL	−1.92E-02	1.48E-02	−1.298		Interaction GBP/FL	−2.18E-03	9.11E-04	−2.394	*
Interaction KO/FL	−2.20E-02	1.48E-02	−1.483		Interaction KO/FL	−2.03E-03	9.28E-04	−2.193	*
Interaction GBP/KO	2.24E-02	1.48E-02	1.514		Interaction GBP/KO	1.61E-03	9.24E-04	1.747	
CUX1 PMZ					CTIP2 PMZ				
FL Injury	−3.53E-03	9.94E-04	−3.550	*	FL Injury	−3.40E-03	8.31E-04	−4.096	*
GBP treatment	1.35E-04	9.14E-04	0.148		GBP treatment	−2.35E-04	7.38E-04	−0.318	
α2δ-1 KO	1.42E-03	9.86E-04	1.438		α2δ-1 KO	7.33E-04	8.27E-04	0.887	
Interaction GBP/FL	3.05E-03	1.13E-03	2.710	*	Interaction GBP/FL	1.09E-03	9.38E-04	1.158	
Interaction KO/FL	1.34E-03	1.14E-03	1.176		Interaction KO/FL	2.20E-03	9.64E-04	2.280	*
Interaction GBP/KO	−2.21E-03	1.14E-03	−1.944		Interaction GBP/KO	1.78E-04	9.65E-04	0.185	
CUX1 MZ					CTIP2 MZ				
FL Injury	−2.79E-03	9.17E-04	−3.047	*	FL Injury	−2.56E-03	9.44E-04	−2.708	*
GBP treatment	−3.86E-05	8.48E-04	−0.046		GBP treatment	−2.08E-04	8.51E-04	−0.244	
α2δ-1 KO	1.19E-03	9.10E-04	1.309		α2δ-1 KO	7.44E-04	9.38E-04	0.794	
Interaction GBP/FL	2.32E-03	1.04E-03	2.232	*	Interaction GBP/FL	2.60E-03	1.07E-03	2.431	*
Interaction KO/FL	1.42E-03	1.05E-03	1.361		Interaction KO/FL	1.96E-03	1.09E-03	1.800	
Interaction GBP/KO	−1.79E-03	1.05E-03	−1.706		Interaction GBP/KO	1.20E-04	1.09E-03	0.110	
Superficial/deep layer area ratio					Synapse number				
FL Injury	5.613	1.523	3.685	*	FL Injury	301.184	78.931	3.816	*
GBP treatment	NA	NA	NA		GBP treatment	4.534	77.715	0.058	
α2δ-1 KO	NA	NA	NA		α2δ-1 KO	−49.436	77.715	−0.636	
Interaction GBP/FL	−3.877	1.413	−2.743	*	Interaction GBP/FL	−195.500	90.606	−2.158	*
Interaction KO/FL	−5.235	1.556	−3.365	*	Interaction KO/FL	−142.822	90.606	−1.576	
Interaction GBP/KO	3.710	2.132	1.740		Interaction GBP/KO	−28.255	90.634	−0.312	
fEPSP % epileptiform					fEPSP area				
FL Injury	0.447	0.131	3.409	*	FL Injury	22.953	6.458	3.554	*
GBP treatment	−0.179	0.132	−1.352		GBP treatment	−1.940	6.553	−0.296	
α2δ-1 KO	−0.177	0.140	−1.265		α2δ-1 KO	−7.069	6.365	−1.111	
Interaction GBP/FL	−0.235	0.148	−1.588		Interaction GBP/FL	−22.600	7.851	−2.879	*
Interaction KO/FL	−0.211	0.147	−1.439		Interaction KO/FL	−6.182	7.719	−0.801	
Interaction GBP/KO	0.283	0.147	1.926		Interaction GBP/KO	15.274	7.839	1.948	

*t value > 1.96 or < −1.96.

## Discussion

Treatment with GBP, a clinically used anticonvulsant and antagonist of α2δ-1, attenuates pathologic changes following experimental neonatal and adult brain insult (Traa et al., 2008; Li et al., 2012; Andresen et al., 2014). α2δ-1 (a calcium channel auxiliary subunit) and its synaptogenic ligand, TSP, are increased following brain trauma, suggesting injury-induced activation of α2δ-1 signaling. Defining the precise role of α2δ-1 in the pathology of brain injury has been difficult, however, as GBP is known to directly bind to both α2δ-1 and α2δ-2, and has indirect effects on other systems (NMDA receptors; Kim et al., 2009), sodium channels (Yang et al., 2009), and protein kinase C (Maneuf and McKnight, 2001; Yeh et al., 2011). Here, we used mice lacking α2δ-1 to establish its role in pathology associated with FL, and to determine the site of action of GBP’s neuroprotective effects. We show, for the first time, that α2δ-1 plays a direct role in cell death, anatomic reorganization, synaptogenesis, astrocytosis, expression of layer-specific cortical markers, and network hyperexcitability in the FL model of cortical malformation. By genetically deleting α2δ-1, we established that it mediates GBP’s neuroprotective effects. Our findings show α2δ-1 has an important role in injury-induced cell death and synaptogenesis, and suggest that α2δ-1 activity may be involved with pathogenesis associated with acquired DCMs.

GBP treatment is neuroprotective in models of cortical deafferenation (Li et al., 2012), neonatal ischemia (Traa et al., 2008), and ischemic brain injury (Williams et al., 2006), likely due to attenuation of TSP/α2δ-1 signaling. Human studies show elevated TSP-1 in plasma after head trauma (Wang et al., 2016), human endothelial cells upregulate TSP-1 after oxidative stress (Ning et al., 2011), and TSP1 and TSP2 are increased in rodent models of stroke (Lin et al., 2003). Likewise, α2δ-1 expression is upregulated in injury models in both the central and peripheral nervous system (Boroujerdi et al., 2008; Li et al., 2012; Andresen et al., 2014), leading to an overabundance of both ligand and receptor. Beyond changes in expression, α2δ-1’s subcellular localization is altered in areas of neuronal death following chemoconvulsive seizures (Nieto-Rostro et al., 2014), suggesting subtle changes in α2δ-1 signaling may be relevant to cell death and epileptogenesis.

How α2δ-1 signaling leads to cell death is not clear, but calcium-dependent caspase-mediated apoptosis, appears to be involved. Loss of α2δ-1 decreases the duration of calcium channel currents in cardiac myocytes (Fuller-Bicer et al., 2009) and calcium channel density (Patel et al., 2013) in sensory neurons. Conversely, over expression of α2δ-1 increases neurotransmitter release following action potential firing in the hippocampus (Hoppa et al., 2012), which could leader to greater excitation. We see two ways in which α2δ-1 might contribute to injury-induced cell death. First, by increasing excitatory synapse formation and enhancing excitation, α2δ-1 activity likely potentiates glutamate-induced excitotoxicity. Second, by enhancing calcium currents and intracellular calcium levels, α2δ-1 may promote caspase activation. Both aspects of α2δ-1 may contribute to lesion-induced cell death, and undoubtedly occur in parallel with other forms of cell death that do not involve α2δ-1. Future studies will be required to identify which aspects of α2δ-1 function lead to injury-induced cell death and if certain cell types are more vulnerable to α2δ-1-mediated cell death.

In addition to driving cell death, FL also induces robust astrocytosis (Bordey et al., 2001; Campbell and Hablitz, 2008; Dulla et al., 2013; Armbruster et al., 2014). Here, we show that α2δ-1 signaling plays an important role in driving reactive astrocytosis. GFAP immunoreactivity was greatly reduced by attenuating α2δ-1 signaling, and no additional effect of GBP was seen in α2δ-1 KO animals. Although astrocytes express α2δ-1 at a low-level, it is much more abundantly expressed by neurons (Zhang et al., 2014). This suggests that decreased astrocyte reactivity may be a secondary effect of reduced cell death and subsequent neuroinflammation, rather than a direct effect on α2δ-1 expressed by astrocytes. How α2δ-1 contributes more broadly to neuroinflammation, macrophage lineage cells, neuro-immune interactions, and cell death signaling warrants future investigation.

α2δ-1 activity following FL also promotes lesion formation and disrupts cortical lamination (shown by CUX1 and CTIP2 staining), in both the MZ and PMZ. Previous reports describe the neuronal architecture of the PMZ as normally laminated. Our results confirm that although gross level changes in cortical structure and total neuron number do not occur in the PMZ, more subtle differences may exist, as indicated by the reduction in the density of in CUX1+ and CTIP2+ neurons. How this change is linked to FL-induced pathologies is unknown. CTIP2 or CUX1 are both transcription factors (Molyneaux et al., 2007; Sansregret and Nepveu, 2008) that control the expression of proteins related to neuronal morphologic development (Arlotta et al., 2005; Li et al., 2010). Whether changes in the density of CUX1+ and CTIP2+ neurons are associated with known changes in synaptic connectivity or neuronal morphology remains to be seen.

In addition to the established role of TSP/α2δ-1 signaling in developmental synaptogenesis, our work also supports a role for α2δ-1 in pathologic synaptogenesis. α2δ-1 KO and *in vivo* treatment with GBP attenuated injury-induced increases in both anatomic and functional synapses, in agreement with previous studies (Li et al., 2012; Andresen et al., 2014). Although functional synapses were not affected by GBP treatment in α2δ-1 KO mice, there was a minor reduction in the abundance of structural synapses. Other α2δ subunits may mediate these effects. α2δ–1, α2δ–2, and α2δ–3 are all widely expressed in the CNS, with each isoform having a distinct distribution (Cole et al., 2005). Genetic deletion of α2δ–1 does not grossly alter the expression of other α2δ isoforms (Fuller-Bicer et al., 2009), however, regional compensation or injury-specific alterations may still exist and be relevant to FL-induced pathologies. GBP can bind to α2δ–2 (Gee et al., 1996) and α2δ–3 has been shown to play a synaptogenic role in drosophila (Kurshan et al., 2009). Taken together, these results suggest that other α2δ isoforms may account for the GBP effect on synapse number in the α2δ-1-KO FL cortex. Additionally, it is unknown whether changes in other calcium channel auxiliary subunits occur following genetic deletion of α2δ-1 or neonatal injury. Also important to note, only vGlut1+ synapses, the predominant vGlut in the cortex, were quantified here. TSP/α2δ-1 signaling drives the formation of vGlut2 containing synapses during normal development (Eroglu et al., 2009). Conversely, GBP treatment reduces injury-induced increases in vGlut1-containing excitatory synapses (Li et al., 2012). Our studies further support a role of α2δ–1 in driving vGlut1-containing synapse formation after injury, in addition to its role in driving vGlut2-containing synapses during development. This suggests injury increases intracortical excitation, mediated by vGlut1-containing synapses (Fremeau et al., 2004). Changes in vGlut2-containing synapses, calcium channel trafficking (Hendrich et al., 2008), and homeostatic plasticity may underlie the discrepancy in the effects of GBP in α2δ–1 KOs on functional versus structural synapses.

Finally, inhibiting α2δ-1 signaling reduced cortical network hyperexcitability after FL. While genetic deletion of α2δ-1 attenuated FL-driven synaptogenesis, similar to GBP treatment, the effects on network hyperexcitability were intermediate to those seen with GBP treatment. Furthermore, GBP treatment had no effect on the residual epileptiform activity in the α2δ-1 KO animals, despite decreasing anatomic synapses. This suggests that constraining excitatory synaptogenesis is not sufficient to completely prevent hyperexcitability in the α2δ-1 KO cortex, and that deletion of α2δ-1 may cause compensatory changes that predispose the cortex to injury-induced network pathology. Genetic deletion of α2δ-1 did not alter the amplitude or kinetics of excitatory currents, which argues against changes in postsynaptic receptors. Therefore, presynaptic changes, via altered voltage-gated calcium channel function, or changes in neuroinflammatory response may promote hyperexcitability in the α2δ-1 KO cortex. Additionally, α2δ-1 KO may cause differences brain size, vascularization, neuronal morphology, and more, which may be relevant to FL-induced pathology.

To examine the robustness of our findings, we employed a linear mixed effect model, a statistical approach used in clinical studies. This approach revealed the effects of FL were extremely robust and the effects of GBP treatment and α2δ-1 KO in FL animals varied by assay, but were largely robust as well. Minor discrepancies between a standard statistical approach and the linear mixed effect model include the effects of GBP treatment and α2δ-1 KO on the expression of layer specific markers and of α2δ-1 KO on network hyperexcitability following FL. This approach illustrated areas that require more study before translational application; overall, however, the model confirmed that α2δ-1 signaling plays a critical role in much of the pathogenesis of the FL model.

Overall, our results show that significant α2δ-1-mediated pathologies occur in the FL model and a growing number of studies suggest that α2δ-1 signaling may contribute to the development of epilepsy. Overexpression of α2δ-1 in control animals leads to epileptiform EEG discharges and behavioral arrests, which are rescued by acute treatment with an anticonvulsant (Faria et al., 2017). The α2δ-1 ligand TSP-1 shows increased genetic variation in patients with idiopathic/genetic generalized epilepsies. Interestingly, TSP-1 mRNA levels are reduced in the ventrobasal thalamus of WAG/RiJ rats, a genetic model absence epilepsy (Santolini et al., 2017). Additionally, human mutations in α2δ-1 have been linked to polymicrogyria (Vergult et al., 2015). Whether cell death plays a role in these cases is unknown. Furthermore, the functional consequences of these mutations are not known, but gain of function mutations could increase calcium currents, increase cell death, and drive pathologic excitatory synaptogenesis. Also, although attenuating α2δ-1 signaling may reduce cell death and network hyperexcitability, it may compromise functional recovery. Reestablishing synaptic connectivity after injury is a critical aspect of rehabilitation and attenuating TSP1/2 signaling after stroke reduces behavioral recovery (Liauw et al., 2008). Care must be taken, therefore, in manipulating TSP/α2δ-1 signaling, as it plays a role in both the adaptive and pathologic response to insult. Given these caveats, parsing apart the relationship between cell death, synaptogenesis, and epileptiform activity will enable us to optimize treatment strategies that target α2δ-1 signaling in neonatal injury and DCMs.

## Synthesis

Reviewing Editor: Alfonso Represa, INSERM Decisions are customarily a result of the Reviewing Editor and the peer reviewers coming together and discussing their recommendations until a consensus is reached. When revisions are invited, a fact-based synthesis statement explaining their decision and outlining what is needed to prepare a revision will be listed below. The following reviewer(s) agreed to reveal their identity: Jean-Bernard Manent, Albert Becker.

Both referees agree that this is a very interesting and well-conducted study, which has been nicely improved after its revision and transfer to eNeuro. They have only minor comments that you can clarify/discuss on the text for improving the manuscript:

1) The authors show clear evidences that neocortical lamination is reorganized after neocortical freeze lesions, and the illustrated images and quantifications are convincing. Such a precise quantification of laminar reorganization with layer makers has never been performed in the freeze lesion model so far. However, based on their results the authors could hardly claim that the modified cell densities of CUX1+ and CTIP2+ cells are linked to changes in markers expression. This would imply that the molecular identity of those cells has been re-specified, and convincingly investigating this issue would certainly require much more work, outside the scope of the present report. I would thus suggest to tone down or remove this statement from the results section (page 14, lines 29-30) and discussion (page 19, paragraph 2).

2) A recent report suggesting that thrombospondin-1 may contribute to the pathogenesis of absence epilepsy in a rat model and in patients was recently published (Santolini et al., Epilepsia. 2017 Sep 15. doi: 10.1111/epi.13898). This report may be included in the cited references, as another study suggesting thrombospondin/alpha2delta1 signaling could contribute to the development of epilepsy.

3) With respect to the freeze-induced lesion size, can preexisting differences in brain size and/or structure, neuronal arborization and synapse numbers as well as distribution in the area subjected to the lesion induction, vessel density and blood brain barrier structure between α2δ-1 KO mice and control littermates entirely be ruled out as factors with relevant impact?

4) Are there compensatory expression alterations of other auxiliary calcium channel subunits in brain tissue in the region where freeze lesions are induced in α2δ-1 KO compared to control mice?

5) Significance statement, last sentence: Gabapentin has α2δ-1 independent ‘anti’-synaptogenic effects.

## Author Response

We would like to thank the editors and reviewers so much for their largely positive and enthusiastic review. The referees bring up a number of important points which we have addressed below. The reviewer's comments are in black, our responses are in blue. All changes made to the text of the manuscript have also been highlighted in blue.

1) The authors show clear evidences that neocortical lamination is reorganized after neocortical freeze lesions, and the illustrated images and quantifications are convincing. Such a precise quantification of laminar reorganization with layer makers has never been performed in the freeze lesion model so far. However, based on their results the authors could hardly claim that the modified cell densities of CUX1+ and CTIP2+ cells are linked to changes in markers expression. This would imply that the molecular identity of those cells has been re-specified, and convincingly investigating this issue would certainly require much more work, outside the scope of the present report. I would thus suggest to tone down or remove this statement from the results section (page 14, lines 29-30) and discussion (page 19, paragraph 2).

We agree with your assessment and have edited the text to remove claims that marker expression has changed. The text in those sections now reads:

Results, page 14-

These results suggest that overall neuronal density is normal in the FL cortex at P28, despite changes in CUX1+ and CTIP2+ cell density.

Discussion, page 19-

α2δ-1 activity following FL also promotes lesion formation and disrupts cortical lamination (shown by CUX1 and CTIP2 staining), in both the MZ and PMZ. Previous reports describe the neuronal architecture of the PMZ as normally laminated. Our results confirm that although gross level changes in cortical structure and total neuron number do not occur in the PMZ, more subtle differences may exist, as indicated by the reduction in the density of in CUX1+ and CTIP2+ neurons. How this change is linked to FL-induced pathologies is unknown. CTIP2 or CUX1 are both transcription factors (Molyneaux et al., 2007; Sansregret and Nepveu, 2008) that control the expression of proteins related to neuronal morphological development (Arlotta et al., 2005; Li et al., 2010). Whether changes in the density of CUX1+ and CTIP2+ neurons are associated with known changes in synaptic connectivity or neuronal morphology remains to be seen.

2) A recent report suggesting that thrombospondin-1 may contribute to the pathogenesis of absence epilepsy in a rat model and in patients was recently published (Santolini et al., Epilepsia. 2017 Sep 15. doi: 10.1111/epi.13898). This report may be included in the cited references, as another study suggesting thrombospondin/alpha2delta1 signaling could contribute to the development of epilepsy.

Thank you for bringing this recent paper to our attention. We have added this reference to the discussion.

Discussion, page 20-

The a2d-1 ligand thrombospondin is altered in patients and animal models of absence epilepsy (Santolini et al., 2017)

We have also taken this opportunity to include another recent paper on the role α2δ-1 in injury-induce epileptogenesis

Takahashi DK, Jin S, Prince DA. Gabapentin Prevents Progressive Increases in Excitatory Connectivity and Epileptogenesis Following Neocortical Trauma. Cereb Cortex. 2017 Jun 26:1-16. doi: 10.1093/cercor/bhx152. [Epub ahead of print]

3) With respect to the freeze-induced lesion size, can preexisting differences in brain size and/or structure, neuronal arborization and synapse numbers as well as distribution in the area subjected to the lesion induction, vessel density and blood brain barrier structure between α2δ-1 KO mice and control littermates entirely be ruled out as factors with relevant impact?

We have not performed extensive characterization of α2δ-1 KO mice in regards to brain size, neuronal arborization, vascularization, or blood brain barrier structure. The reviewers are correct that we cannot exclude the possibility that these changes may be relevant. Our current study was optimized to detect differences post-injury and therefore may have missed subtle differences in the uninjured animal. We have added the line:

Discussion, page 12-

Additionally, α2δ-1 KO may cause differences brain size, vascularization, neuronal morphology, and more, which may be relevant to FL-induced pathology.

4) Are there compensatory expression alterations of other auxiliary calcium channel subunits in brain tissue in the region where freeze lesions are induced in α2δ-1 KO compared to control mice?

The reviewer brings up an interesting report that we have not addressed in this study. In two other recent reports, the loss of α2δ-1 did not affect the levels of α2δ-2, -3 and -4 in the cortex (Fuller-Bicer et al., Am J Physiol Heart Circ Physiol., 2009) or in dorsal root ganglion cells at baseline or following peripheral nerve injury (Patel et al., Neurobiol Dis., 2013). To our knowledge, no studies have examined the expression of other auxiliary calcium channel subunits in α2δ-1 KO animals. If there are compensatory changes in these isoforms following freeze lesion, it does not appear to preclude the protective effects of α2δ-1 genetic deletion on exuberant synaptogenesis, circuit reorganization or cell death. We plan to further address this issue in our future work. We are currently examining mRNA expression of a panel of neuronal, injury, and inflammation related genes as we explore how α2δ-1 signaling might contribute to cell death and other FL-associated pathologies. To address this point, we have edited the following section to the discussion:

Discussion, page 19-

Genetic deletion of α2δ-1 does not grossly alter the expression of other α2δ isoforms (Fuller-Bicer et al., 2009), however, regional compensation or injury-specific alterations may still exist and be relevant to FL-induced pathologies. GBP can bind to α2δ-2 (Gee et al., 1996) and α2δ-3 has been shown to play a synaptogenic role in drosophila (Kurshan et al., 2009). Taken together, these results suggest that other α2δ isoforms may account for the GBP effect on synapse number in the α2δ-1 1-KO FL cortex. Additionally, it is unknown whether changes in other calcium channel auxiliary subunits occur following genetic deletion of α2δ-1 or neonatal injury.

5) Significance statement, last sentence: Gabapentin has α2δ-1 independent ‘anti’-synaptogenic effects.

Thank you for pointing this out. We have corrected the text in this line of the significance statement.
